# Graphene quantum dots as the electrolyte for solid state supercapacitors

**DOI:** 10.1038/srep19292

**Published:** 2016-01-14

**Authors:** Su Zhang, Yutong Li, Huaihe Song, Xiaohong Chen, Jisheng Zhou, Song Hong, Minglu Huang

**Affiliations:** 1 State Key Laboratory of Chemical Resource Engineering, Beijing Key Laboratory of Electrochemical Process and Technology for Materials, Beijing University of Chemical Technology, Beijing, 100029, P. R. China

## Abstract

We propose that graphene quantum dots (GQDs) with a sufficient number of acidic oxygen-bearing functional groups such as -COOH and -OH can serve as solution- and solid- type electrolytes for supercapacitors. Moreover, we found that the ionic conductivity and ion-donating ability of the GQDs could be markedly improved by simply neutralizing their acidic functional groups by using KOH. These neutralized GQDs as the solution- or solid-type electrolytes greatly enhanced the capacitive performance and rate capability of the supercapacitors. The reason for the enhancement can be ascribed to the fully ionization of the weak acidic oxygen-bearing functional groups after neutralization.

Because of their unique two-dimensional feature, large specific surface area, and favorable electronic properties, graphene-based materials are promising candidates for the electrode materials of supercapacitors^1^. Recently, several novel capacitor devices including microsupercapacitors^2^, all-solid-state supercapacitors^3^,^4^, and wearable supercapacitors have been developed on the basis of graphene materials^5^,^6^,^7^,^8^. Graphene oxide (GO) is a crucial derivation of graphene^9^,^10^. Commonly, GO is prepared using chemical oxidation of graphite powder^11^. GO performed high ionic conductivity due to the decorated oxygen-bearing functional groups such as -OH, -C = O, and -COOH on its surface. In addition, a large amount of H_3_O^+^ can be ionized from its acidic functional groups such as -OH and -COOH. However, sp^3^ hybridized carbon atoms introduced by functional groups also destroy the aromatic conjugated structure of graphene flake, resulting in the electrical insulation of GO. Based on these properties, Ajayan *et al.* proposed a simple laser writing route fabricating shape-controlled micro-supercapacitors on graphite oxide film^2^. In these devices, GO functioned as both a solid-state electrolyte and a separator.

Graphene quantum dots (GQDs) are another novel graphene derivation which attracted considerable attention recently^12^,^13^,^14^. Recently, Yan *et al.* proposed that GQDs can be used as high-performance electrode materials for microsupercapacitors^15^,^16^. Three dimensional graphene-GQD hydrogels prepared by Qu *et al.* demonstrated high capacitive and cyclic performance for supercapacitors^17^.GQDs derived from chemical oxidation of carbonaceous precursors can be considered extremely small GO debris with a sufficient number of acidic oxygen-bearing functional groups^18^,^19^. Herein, we propose that GQDs can be used as the solution- and solid- type electrolytes for supercapacitors. To the best of our knowledge, this property has rarely been investigated. Moreover, the ionic conductivity and ion-donating ability of GQDs could be considerably improved by simply neutralizing the weak acidic oxygen-bearing functional groups by using KOH. When the neutralized GQDs (NGQDs) were used as the solution- or solid- type electrolytes, the capacitive and cyclic performance of the supercapacitors was greatly enhanced. Our findings can provide new insights into applying GQDs and developing graphene based solid state supercapacitors and could help to: (1) clarify the ionic conductivity of GO, (2) broaden the potential applications of GQDs, and (3) develop a new type solid-state electrolyte for supercapacitors.

## Experimental

### Material preparation

All the chemical agents we used were analytically pure without further purification. All the water used was 18.2 MΩ cm.

In this study, graphite oxide was prepared by using a modified Hummers method according to Ref. 20. We used graphene nanosheets (GNSs) as an exemplified electrode material. The GNSs were prepared by applying thermal expansion of the as-prepared graphite oxide powder at 1050 °C for 30 s in a muffle oven^20^.

To prepare the GQDs, 1.0 g of the as-prepared graphite oxide, 30 mL of the concentrated HNO_3_ (38 wt. %), and 90 mL of the concentrated H_2_SO_4_ (98 wt. %) were carefully added to a 250 mL three-neck round-bottom flask and refluxed at 85 °C for 48 h. After cooling to room temperature, the product was diluted into 600 mL of the deionized water (This step was processed in a fuming cupboard because a large amount of NO_2_ gas would be released). The product was centrifuged at 4000 rpm for 20 min. A highly acidic GQD suspension was collected from the supernatant. To remove the excess acids, the suspension was diluted into ca. 2000 mL of deionized water and further dialyzed using a dialysis bag (molecular weight cut off = 3500 Da) until the pH value of the outside water was 7 (tested after standing for 6 h). The GQD suspension was concentrated by using a rotary evaporator at 50 °C. The GQD powder was obtained by drying the concentrated GQD suspension at 50 °C in vacuum oven.

To prepare the GQD solution electrolyte, the GQD powder was redispersed into deionized water at a concentration of 40 mg mL^−1^. The NGQD solution electrolyte was prepared by neutralizing the GQD solution electrolyte using KOH solution (6 M). The pH value of the GQD solution electrolyte was carefully modulated to 7.

### Characterization

Fourier-transform infrared (FT-IR) spectroscopy was conducted using a Nicolet iS50 infrared spectroscopy instrument. The ^13^C solid-state nuclear magnetic resonance (^13^C SSNMR) spectrum was recorded on a Bruker AV300 system at a frequency of 12 kHz. X-ray photoelectron spectroscopy (XPS) was conducted using an Escalab 250 system using a monochromatic AlKα (1486.6 eV) X-ray source with 30 eV pass energy in a 0.5 eV step over an area of 650 × 650 μm. The micromorphology of the samples was characterized using high-resolution transmission electron microscopy (HRTEM, JEM-2100F). The samples were dispersed in ethanol and dropped on an ultra-thin carbon supported film.

### Electrochemical measurements

The electrochemical performance was measured using a two-electrode symmetric system assembled using LIR 2032 cells. The electrodes were prepared by coating GNSs (90 wt. %) and poly (vinylidene difluoride) (10 wt. %) on nickel foams and compressing them into flat sheets. All the supercapacitor devices were assembled using the same components and electrodes under the same conditions. The as-prepared GQD and NGQD solutions were used as the liquid electrolytes. Cellulose paper (Nippon Kodoshi Corporation) was used as the separator. To prepare solid-state GQD or NGQD electrolytes, ca. 0.2 mL of the GQD or NGQD solution electrolyte was doped on the cellulose paper and dried at 60 °C in an air atmosphere to fabricate GQD or NGQD solid-state films. Humidity played a crucial role in the ionic conductivity of the GO film^2^. In this study, when the GQD or NGQD films were used as the solid-state electrolyte, we added 10 μL of deionized water to the cells during assembly to ensure a humid environment. The supercapacitors were galvanostatically charged and discharged in a voltage range from −0.3 to 0.3 V. The cyclic voltammetry (CV) and electrochemical impedance spectroscopy (EIS) were conducted on an electrochemical workstation (CHI660B). The supercapacitors were initially maintained at 0 V for CV and EIS measurements. The impedance spectra were obtained by applying a sine wave with an amplitude of 5.0 mV over a frequency range of 10 kHz to 1 Hz.

## Results and Discussion

**Figure 1:**
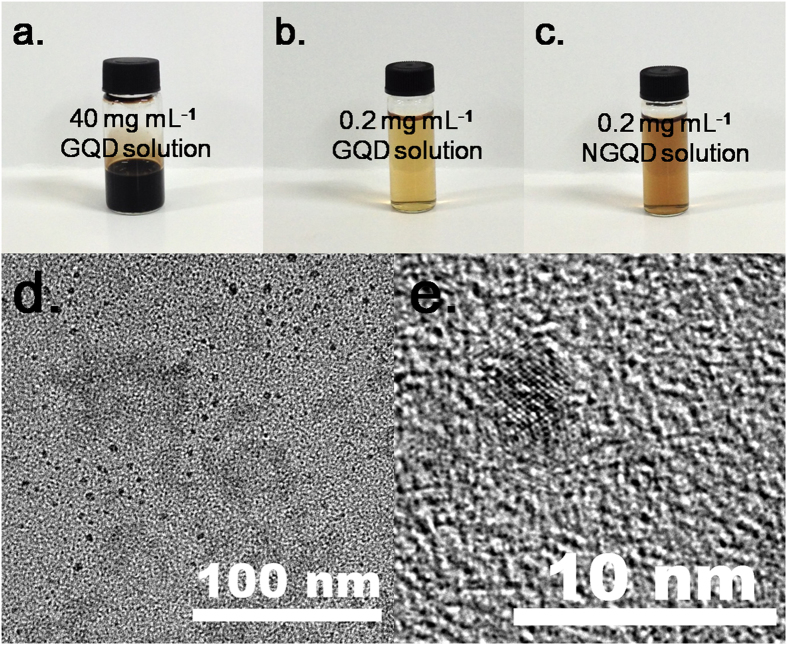
Photographs of (**a**) 40 mg mL^−1^ GQD solution, (**b**) 0.2 mg mL^−1^ GQD solution, and (**c**) 0.2 mg mL^−1^ NGQD solution; (**d**) and (**e**) HRTEM images of the GQDs.

Figure 1(a–c) shows the GQD solution (40 mg mL^−1^), diluted GQD solution (0.2 mg mL^−1^), and diluted NGQD solution (0.2 mg mL^−1^), respectively. The GQDs with a sufficient number of oxygen-bearing functional groups showed exceptionally high water dispersibility. No precipitation was observed even after the GQD solution was maintained for 3 months. The color of the GQD dispersion changed from light orange to brown after KOH neutralization. This phenomenon was also observed in the alkaline GO solution^21^. Figure 1 d and e show the HRTEM images of the GQDs. The diameters of the obtained GQDs ranged from 2 to 5 nm.

**Figure 2:**
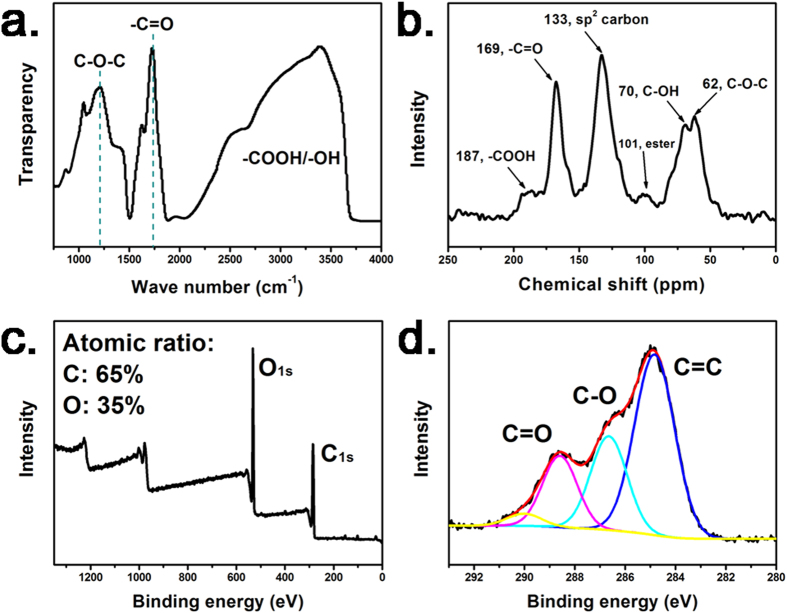
(**a**) FT-IR, (**b**) ^13^C SSNMR, (**c**) XPS, and (**d**) C1s spectra of the obtained GQDs.

The FT-IR and ^13^C SSNMR spectra of the GQDs displayed in Fig. 2a,b indicated that a sufficient number of oxygen-bearing functional groups such as C-O-C, -C-OH, -C = O, and -COOH were decorated on the GQDs^22^,^23^,^24^. The elemental atomic ratios of the GQDs are presented in the XPS spectrum in Fig. 2c. Tour *et al.* proposed that the acidity of GO originates from the gradual interaction of surface oxygen-bearing functional groups and water molecules^24^,^25^. Rourke *et al.* reported that GO can be considered the high oxidative carbonaceous debris attached to low oxidative large platelets^21^. Accordingly, GQDs can also be considered high oxidative GO debris. When GQDs are dispersed in water and maintained for a certain time, hydrolysis can occur on ester and other oxygen-bearing functional groups, resulting in the formation of acidic functional groups such as -COOH and -OH^2^. We observed that when GQD solution was dialysis for several days, the water out of the dialysis bag was neutral, but the GQD solution was still acidic. The pH value of the dialyzed GQD solution (0.2 mg mL^−1^) is ca. 3.8. The acidity of GQDs enables it to release H_3_O^+^. Thus the GQD solution and solid-state films (in a humid environment) can be used as the electrolytes for supercapacitors.

**Figure 3:**
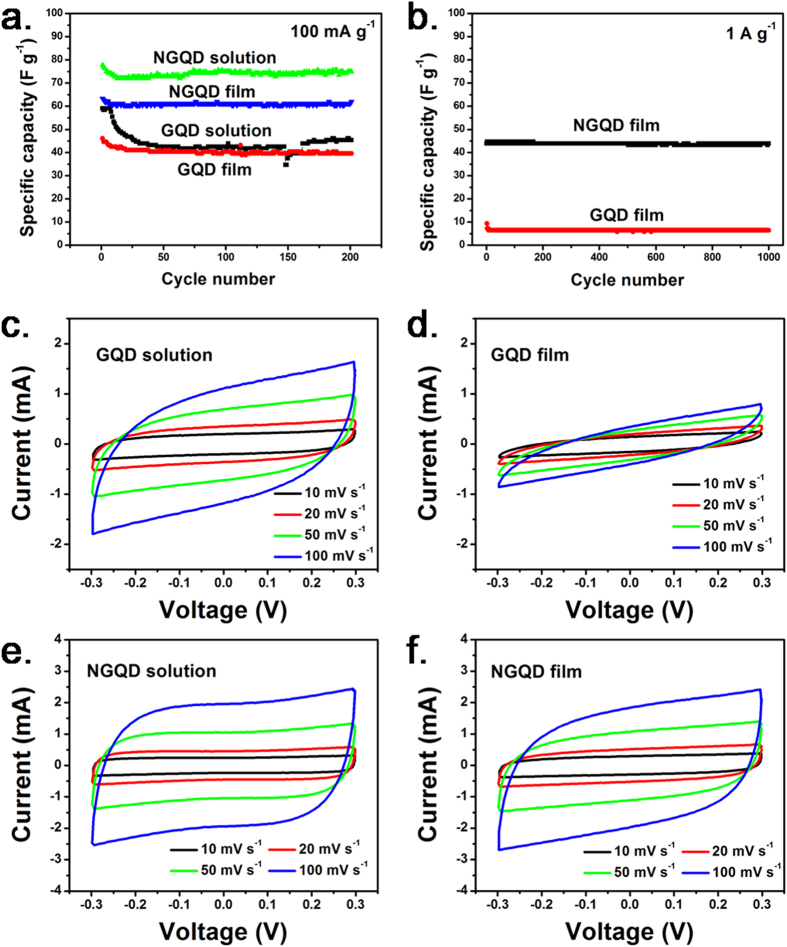
(**a, b**) Galvanostatic charge-discharge curves and (**c–f**) CV curves of the assembled supercapacitors using GNSs as the exemplified electrode material and GQD or NGQD solutions, or GQD or NGQD solid-state films as the electrolytes. The as-used electrolytes were marked in each of the figures.

**Figure 4:**
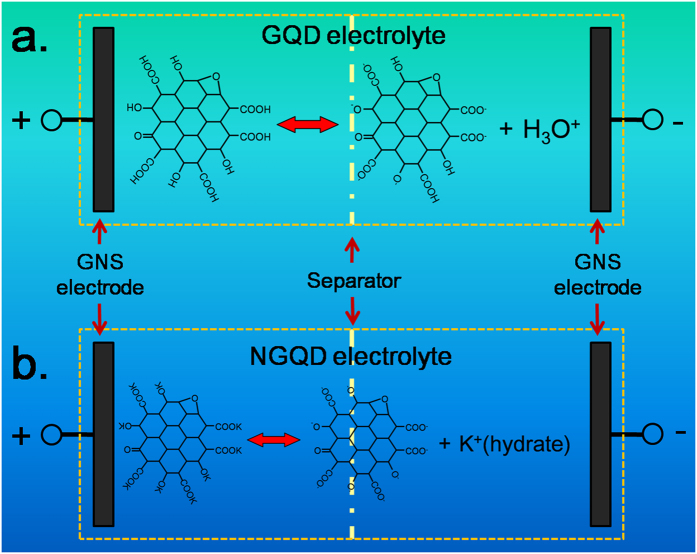
mechanism schematic of supercapacitors using (**a**) the GQD electrolyte and (**b**) the NGQD electrolyte.

Figure 3 shows the electrochemical performance of the assembled supercapacitors. GNSs were used as the exemplified electrode materials, and the obtained GQD- or NGQD- solutions or solid-state films were used as the electrolytes. At a current density of 100 mA g^−1^, the supercapacitors using the GQD solution- and film-type electrolytes show the similar specific capacity of ca. 42 F g^−1^ (Fig. 3a). The CV curves of the supercapacitors using the GQD solution (Fig. 3c) and solid-state films (Fig. 3d) were approximately rectangular without obvious redox peaks. This indicated the electrochemical double-layer capacitive performance of the GNS electrodes in which the energy storage mechanism is based on reversible ion adsorption-desorption on its surface^26^,^27^,^28^. This phenomenon is similar to that observed when using KOH solution as the electrolyte^29^. However, the rectangular CV curves of the supercapacitors using the GQD solution and film were distorted at a high scan rate, particularly when the GQD films were used^30^. Although GQDs can release hydronium ion under humid conditions, the oxygen-bearing functional groups on GQDs such as -COOH and -OH are mainly weak acidic. The GQD can be considered as weak organic acid in this case. The incomplete ionization of oxygen-bearing functional groups leads to the low ion concentration and ionic conductivity of GQD electrolytes. Furthermore, increasing ion adsorption on the surface of a GNS electrode with increasing applied voltage causes the consumption of ions in the electrolyte. To counteract the consumption, the weak acidic oxygen-bearing functional groups on GQDs must be ionized further. The kinetics of this process also results in large electrochemical polarization of the electrode at high scan rate, and is considered the main reason for the distortion of the CV curves. The mechanism schematic is shown in Fig. 4a.

In this study, when the GQDs were neutralized by KOH, the specific capacities of the supercapacitors were substantially enhanced (Fig. 3a). At the current density of 100 mA g^−1^, the supercapacitors assembled using the NGQD solution and film electrolytes showed the capacity of ca. 70 F g^−1^ and 60 F g^−1^, respectively. Moreover, the rate capability of the supercapacitors using the NGQD film was considerably higher than that of those using the GQD film. At the current density of 1 A g^−1^, the specific capacity of the supercapacitors using the NGQD film was ca. 45 F g^−1^, whereas it yielded only ca. 6 F g^−1^ for the GQD film (Fig. 3b). The CV curves shown in Fig. 3c,f were rectangular even at a high scan rate. The maintained shapes of the CV curves indicated that after neutralization, the conductivity and ion-donating ability of the NGQD solution- or solid-type electrolytes were markedly increased^27^,^30^.

**Figure 5:**
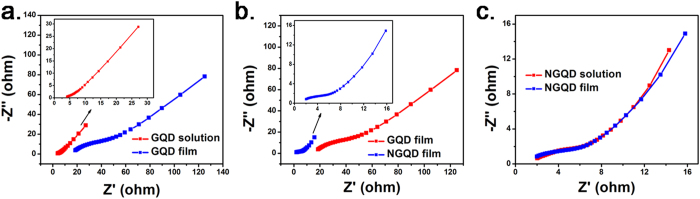
EIS curves of the supercapacitors assembled using (**a**) the GQD solution and solid-state films, (**b**) GQD and NGQD solid-state films, and (**c**) NGQD solution and solid-state films as the electrolytes.

To further study the kinetic process of the obtained supercapacitors, EIS was conducted; the spectra are illustrated in Fig. 5. The intersection between the curve and the horizontal axis represents the total electric resistance of the devices. This electric resistance is strongly related to (1) ion migration in separators and electrode materials, and (2) the resistances of electrode materials, cell shells, and electrolytes. Because all the supercapacitor devices in this study were assembled using the same components and electrodes under the same conditions, the electric resistance could be considered to reflect the electric resistance of the electrolytes. The diameter of the semicircle at high frequency is related to the charge transfer resistance between electrode materials and the electrolyte, and the tail slope at low frequency is related to the ionic diffusion rate in the electrolyte^29^,^31^,^32^. As shown in Fig. 5a, the supercapacitors using the GQD solution electrolyte showed an electric resistance of ca. 3.8 Ω, which is lower than that of the supercapacitors using the GQD solid state electrolyte (ca. 18.6 Ω). The tail slope was 1.28 for the GQD solution electrolyte and 0.87 for the GQD solid electrolyte. This indicated that the ion diffusion rate of the GQD solution electrolyte was substantially higher than that of the GQD solid electrolyte. Water plays a vital role in the GQD electrolyte because (1) the acidic functional groups in GQDs originate from the hydrolysis of oxygen-bearing groups with water molecules^24^,^25^, and (2) GQDs can be considered weak acid; thus, water is required to facilitate their ionization. Hence we deduced that the GQD solution electrolyte exhibits considerably higher ion diffusion rate and conductivity than those of the GQD solid electrolyte. After neutralization (Fig. 5b), the electric resistance of the supercapacitors using the NGQD solid electrolyte (ca. 1.8 Ω) was markedly lower than that of those using GQD solid electrolyte (ca. 18.6 Ω) and even the GQD solution electrolyte (3.8 Ω). We propose that KOH neutralized GQDs can be considered a type of salt (Fig. 4b). These NGQDs almost fully ionize in a humid environment, resulting in considerable improvement of the ionic conductivity and ion-donating ability of the GQDs under humid conditions. The supercapacitors assembled with the NGQD solution and film electrolytes performed similarly to the kinetic process (Fig. 5c), indicating that NGQDs can be used as both solid and liquid electrolytes for supercapacitors. Moreover, the tail slope in the EIS of the NGQD solution- and solid-type electrolytes is 1.12, indicating the ion diffusion rate of the NGQD electrolyte is higher than that of the GQD solid electrolyte (tail slope 0.87) because of the increased ion concentration. However, it was lower than the ion diffusion rate in the GQD solution electrolyte (tail slope 1.28), likely because diameter of the hydrated K^+^ is longer than that of the hydronium ion. When a common KOH solution (6 M, ca. 330 mg mL^−1^) was used as the electrolyte for GNS based supercapacitors, the specific capacitance is ca. 110 F g^−1^ at the current density of 100 mA g^−1^ and ca. 86 F g^−1^ at 1 A g^−1^ 26. The capacitance was almost two times higher than that using the NGQD electrolytes. We determined that highly concentrated KOH electrolyte can provide (1) higher conductivity and charge carrier density, and (2) superior ion mobility compared with NGQD electrolytes. But we believed that our findings can broaden the applications of GQDs and provide new insights into designing solid-state electrolytes.

## Conclusion

In this study, GQDs were prepared through chemical oxidation of graphite oxide; and GNSs as exemplified electrode materials, were prepared through thermal expansion of graphite oxide powder. Because of their conductivity and ion-donating ability, the solution- and solid-type GQDs could be used as electrolytes for supercapacitors. Moreover, when the GQDs were neutralized by KOH, both the conductivity and ion-donating ability were considerably enhanced. The supercapacitors using the solution- and solid-type NGQD electrolytes demonstrated markedly superior capacitive and rate performance compared with those using GQD electrolytes. The reason for the enhancement can be ascribed to the fully ionization of the weak acidic oxygen-bearing functional groups after neutralization.

## Additional Information

**How to cite this article**: Zhang, S. *et al.* Graphene quantum dots as the electrolyte for solid state supercapacitors. *Sci. Rep.* **6**, 19292; doi: 10.1038/srep19292 (2016).
